# Chronic pain in a modern virally suppressed HIV cohort is associated with disability and poorer mental health

**DOI:** 10.1038/s41598-026-52912-x

**Published:** 2026-05-13

**Authors:** Ronald J. Ellis, Robert K. Heaton, J. Hampton Atkinson, Murray B. Stein, Crystal Wang, Tyler R. Bell, Andrew H. Miller, David Grelotti, David Moore

**Affiliations:** 1 Department of Psychiatry, University of California San Diego, San Diego, CA 92093 USA; 2 Department of Neurosciences, University of California San Diego, San Diego, CA 92093 USA; 3 Department of Psychiatry and Behavioral Sciences, Emory University, Atlanta, GA 30322 USA; 4 University of California, San Diego, 220 Dickinson Street, Suite B, San Diego, CA 92103 USA

**Keywords:** Depression, Mood, Chronic pain, Neuropathic pain, Opioid use, HIV infections, Comorbidities, Pain

## Abstract

Chronic pain (CP) is common among people with HIV (PWH), yet its prevalence and associated factors in those receiving modern, virally suppressive antiretroviral therapy (ART) are not well understood. This prospective observational study compared CP frequency and associated outcomes between PWH and people without HIV (PWoH). Participants (40 PWH, 23 PWoH) completed a questionnaire assessing daily pain lasting more than three months. Additional data included pain intensity, interference with daily activities, opioid use, and depressed mood (Beck Depression Inventory-II), as well as HIV clinical markers and comorbidities. Groups were demographically similar; all PWH were virally suppressed, with a mean HIV duration of 27.0 years, median nadir CD4 count of 173 cells/μL, and median current CD4 count of 644 cells/μL. CP was significantly more frequent in PWH (60%) than in PWoH (22%; OR = 5.40, 95% CI [1.67, 17.50]; *p* = 0.003). Among PWH, CP was associated with greater daily activity interference, higher opioid use, and increased neuropathic pain symptoms. PWH with CP also had higher BDI-II scores, indicating worse mood. These findings suggest that CP remains prevalent and disabling among PWH despite effective modern-day ART, underscoring the need for targeted pain assessment and management in this population.

**Supplementary Information:**

The online version contains supplementary material available at 10.1038/s41598-026-52912-x.

## Introduction

Chronic pain (CP) is more common among people with HIV (PWH; 39–85%) than the general population (20–30%) and remains a major contributor to morbidity despite advances in antiretroviral therapy (ART)^1–5^. CP is a frequent and debilitating condition in the general population, with significant implications for quality of life^1^. Despite this, it remains understudied in PWH, as highlighted in a recent publication from the Global Task Force on Chronic Pain in People with HIV^1^. Although several recent reviews summarize the field^6–8^, there is limited primary data examining CP frequency, sources, and correlates in virally-suppressed PWH on modern ART. Another issue unique to PWH is the frequent co-occurrence of multiple CP disorders^1–3^. CP has also been linked to treatment adherence^6,9^, retention in care^7,10^, and virologic outcomes^11^. These gaps remain clinically important given the growing number of older PWH living longer with chronic comorbidities^12^.

The need to better characterize CP in contemporary, virally-suppressed PWH is underscored by its associations with peripheral neuropathy and other comorbidities that are common in this population^13^. Yet CP in PWH is often underdiagnosed and undertreated^14^. Barriers include limited provider familiarity with HIV-specific pain syndromes, restricted access to specialty pain services, and the cost or unavailability of effective medications^15,16^. Management is further complicated by high rates of substance use and opioid use disorders among PWH^17–19^, which may reflect attempts to self-manage inadequately treated pain.

Depression is another highly prevalent comorbidity in PWH and frequently co-occurs with CP. Although the strong link between CP and depressed mood is well documented in the general population^18,20,21^, these relationships are less well studied in PWH. Shared biological and behavioral pathways—including chronic inflammation, altered reward processing, and HIV-related neurobiological changes—may amplify these associations in PWH^22–24^. Socio-environmental factors such as social isolation, stigma, and financial hardship, which disproportionately affect PWH, may further increase vulnerability to both pain and depressed mood^25^. However, empirical data directly evaluating these interactions in virally-suppressed cohorts remain sparse.

Despite increasing recognition of the importance of CP in aging PWH, major knowledge gaps persist. Few studies have used standardized, IASP/ICD-11–aligned approaches to quantify pain prevalence and characteristics in virally-suppressed PWH, or have compared PWH with demographically similar people without HIV (PWoH) assessed using identical measures. Furthermore, the ways in which pain type (e.g., neuropathic vs. musculoskeletal), comorbid conditions, and pain-related interference differ by HIV serostatus are not well documented.

The objective of this study was therefore to characterize CP in a modern cohort of virally-suppressed PWH, compare it to CP in demographically comparable PWoH, and evaluate its associations with pain type, medical characteristics, mental health, and daily functioning. We hypothesized that CP would be more common in PWH than in PWoH and conducted exploratory analyses to identify clinical correlates and functional impacts of CP in this contemporary HIV cohort.

## Methods

### Participants

The 63 participants (40 PWH, 23 PWoH) included in this analysis were consecutive individuals evaluated at the UCSD HNRC between January 2022 and May 2023, after a standardized CP questionnaire was introduced for all participants. PWH and PWoH were prospectively recruited from the same local community and underwent identical assessments. All participants met HNRC eligibility criteria (age ≥ 21 years, with or without HIV infection documented serologically). No additional sampling or matching procedures were applied. Demographic comparability between groups was confirmed in Table 2. In 2022, in response to increasing community concern and growing research evidence about the significant impact of CP on people with HIV, we added a CP assessment for all participants. Inclusion criteria were age 21 years or over, and with or without HIV infection documented by serologic testing. Potential participants were excluded if they could not participate in the testing due to a variety of conditions such as blindness, deafness, ongoing psychosis, intoxication, or active CNS processes such as brain cancer or opportunistic disease. Such individuals were judged unlikely to contribute meaningful data. Relevant sociodemographic variables, such as age, sex, education, and socioeconomic status, were collected for all participants. The study was approved by the local Ethics Committee of the University of California, San Diego, and was conducted in accordance with the Declaration of Helsinki. All participants gave informed consent for their participation in the study.

### Measures

Multiple measures were used in our study. Full details are provided in Supplementary Material.

*Chronic pain questionnaire* CP was assessed using a structured questionnaire that operationalizes IASP/ICD-11 diagnostic criteria^26^. CP was defined as pain occurring daily or almost daily for ≥ 3 months. Participants endorsing CP completed items assessing pain intensity (0–100 scale), interference with daily activities, medication use, and pain source categories. Following IASP/ICD-11 conventions, we used “pain source” rather than “pain location” to capture clinically meaningful distinctions across musculoskeletal, postsurgical, post-traumatic, neuropathic, and back/leg pain, which are especially relevant in HIV-associated pain^13^. Full item wording, scoring rules, and mapping to IASP/ICD-11 are provided in Supplementary Material.

*Depressed mood and other psychiatric characteristics* Current depressive symptoms were assessed using the Beck Depression Inventory–II (BDI-II)^27^, with reference to validated subscale structures capturing cognitive, somatic, affective, and apathy symptoms^28–30^. Anxiety symptoms were measured with the Overall Anxiety Severity and Impairment Scale (OASIS), a validated 5-item measure of anxiety-related severity and functional impairment^31^. Lifetime psychiatric and substance use disorders were determined using the Composite International Diagnostic Interview (CIDI)^32^, a structured assessment administered by trained interviewers.

*Medical characteristics* HIV diagnosis and clinical monitoring followed standard procedures, including ELISA with Western blot confirmation, CD4 + T-cell counts, routine laboratory panels, and plasma HIV RNA quantification (LLQ = 50 copies/mL). HIV viral load was categorized as detectable vs. undetectable using this threshold. Substance use history included self-reported opioid use and urine toxicology screening for opioids, amphetamines, cannabis, and cocaine; opioid use was classified as present if either self-report or toxicology was positive. Detailed medical and neurological histories, antiretroviral exposure, and comorbidities were obtained through structured clinician-administered interviews. Because neurotoxic NRTIs (stavudine [d4T], didanosine [ddI]) are associated with painful neuropathy, their impact was specifically evaluated. Expanded laboratory methods and coding rules are described in Supplementary Material.

*Clinical assessment of neuropathy and neuropathic pain* Sensory polyneuropathy and distal neuropathic pain (DNP) were characterized using standardized neurological examinations and validated procedures^33,34^. Examinations assessed distal vibration, sharpness and touch loss, and ankle reflexes. DNP was defined as burning, aching, or shooting pain in the distal legs and feet and classified into severity categories based on clinician-rated criteria^33,34^. Full examination procedures, grading criteria, and reliability information are provided in Supplementary Material.

*Quality of life* Quality of life was measured using the Medical Outcomes Study HIV Health Survey Short Form-36 (MOS-HIV SF-36)^35^, which yields summary indices for physical and mental functioning and nine domain scores.

*Sleep quality* Sleep quality and disturbances over the past month were assessed with the Pittsburgh Sleep Quality Index (PSQI)^36^. The PSQI provides a global sleep quality score based on seven component scores.

*Social functioning* Social functioning and satisfaction were measured using the NIH Toolbox Social Satisfaction Factor Score, which assesses emotional support, instrumental support, friendship, loneliness, and rejection^37,38^.

*Activities of daily living* Instrumental activities of daily living (IADLs) were assessed using an adapted Lawton–Brody scale^39^. Participants rated whether they required more assistance with 16 everyday tasks compared to their prior best functioning; scores were summed to derive total IADL complaints.

### Statistical methods

Statistical analyses were performed using JMP Statistical Software (JMP® Student Edition 18.2.0). Demographic and clinical characteristics were summarized using means, standard deviations, medians, interquartile ranges, and percentages as appropriate. The primary analysis compared CP prevalence between PWH and PWoH using Fisher’s exact test; ORs and 95% confidence intervals were calculated to quantify effect sizes. Secondary analyses examined associations between CP and clinical measures using Cohen’s d for continuous variables and Cramér's V for categorical variables, both with bootstrapped 95% CIs (10,000 iterations). Logistic regression was used for binary outcomes. For the BDI-II subscale analyses, false discovery rate (FDR) correction was applied using the Benjamini–Hochberg method to account for multiple comparisons; effect sizes were estimated with bootstrapped 95% CIs. A focused set of univariate and covariate-adjusted (for age and sex) multivariable logistic models was fit within PWH to evaluate whether nadir CD4, current CD4, and HIV duration were associated with CP. To characterize potential bias from missing BDI-II data, we compared participants with and without complete BDI-II data on key demographic, clinical, and pain variables using Cohen’s *d* effect size for continuous variables and Cramér's V for categorical variables.

## Results

### Participants

We evaluated 63 people, 40 PWH and 23 PWoH. Table 1 summarizes the demographic characteristics by HIV serostatus and HIV disease and treatment status of PWH; none of the demographic differences were statistically significant. Among PWH, the mean duration of HIV infection was 27.0 (9.46) years, the nadir CD4 + T lymphocyte count was 173, and the current CD4 + was 644; 100% took ART, and all were virally suppressed. Table 2 compares the demographic and HIV disease and treatment characteristics of PWH without CP (n = 16) and with CP (n = 24). Age, sex, and race/ethnicity were unrelated to CP. None of the HIV disease or treatment variables differed according to CP.

**Table 1:** Demographic and clinical characteristics of people living with and without HIV.

	Statistic	PWH (n = 40)	PWoH (n = 23)	*P*
Demographics				
Age	M (SD)	61.3 (10.5)	57.5 (17.1)	0.346
Sex, female—N (%)	n (%)	9 (23.7%)	9 (45%)	0.099
Education—mean (SD)	M (SD)	14.1 (2.39)	15.1 (2.37)	0.110
Race/Ethnicity				0.693
Black	n (%)	9 (26.5)	4 (22.2)	–
Hispanic	n (%)	6 (15.8)	4 (20)	–
Non-Hispanic White	n (%)	22 (64.7)	12 (66.7)	–
HIV Disease and Treatment				
Estimated Duration of HIV	Mean (SD)	27.0 (9.46)	–	–
Nadir CD4* count	Median (IQR)	173 (17, 323)	–	–
Current CD4	Median (IQR)	644 (488, 786)	–	–
On ART	n (%)	40 (100%)	–	–
Virally suppressed	n (%)	40 (100%)	–	–

*PWH* people with HIV, *PWoH* people without HIV.

*CD4 + T lymphocytes/µl (median (IQR)).

**Table 2:** Demographic and clinical characteristics of people with HIV according to CP status.

	Statistic	No CP (n = 16)	CP (n = 24)	*p*
Demographics				
Age	M (SD)	58.5 (11.5)	62.1 (9.1)	0.301
Sex, female	n (%)	2 (12.5%)	7 (30.4%)	0.212
Years of education	M (SD)	14.5 (2.85)	13.8 (2.07)	0.545
Race/Ethnicity				0.421
Black	n (%)	2 (12.5%)	7 (29.2%)	–
Non-Hispanic White	n (%)	10 (62.5%)	13 (54.2%)	–
Hispanic	n (%)	3 (18.8%)	3 (12.5%)	–
Other	n (%)	1 (6.25%)	1 (4.17%)	–
HIV Disease and Treatment				
Estimated Duration of HIV	Median (IQR)	31.6 (17.8, 35.6)	27.7 (21.7, 34.7)	0.481
Nadir CD4 + T lymphocytes/ul	Median (IQR)	190 (16, 338)	57 (18, 200)	0.240
Current CD4 + T lymphocytes/ul	Median (IQR)	700 (493, 853)	618 (463, 740)	0.395

*CP* chronic pain, *PWH* people with HIV, *PWoH* people without HIV.

In focused univariate and multivariable logistic models within PWH, nadir CD4, current CD4, and HIV duration were not significantly associated with CP (Supplemental Table 1). Age and sex were likewise non-significant in the multivariable model.

### Frequency of chronic pain and associated characteristics

CP frequency was significantly higher in PWH (60%) than in PWoH (22%, OR = 5.40, 95% CI [1.67, 17.50]; Fisher’s exact *p* = 0.003). Among those who reported CP, the average pain intensity did not differ by HIV status: ranged from 10 to 90 out of 100 (0 = no pain; 100 = worst possible pain) (mean ± SD 53.1 ± 22.6) for PWH versus 40 to 65 (51 ± 11.4) for PWoH (d = 0.11). CP interfered more with daily activities among PWH (Not at all 0.0%, A little 21.4%, Somewhat 35.7%, Quite a bit 14.3%, Very much 28.6%) than PWoH (33.3%, 66.7%, 0.0%, 0.0%, 0.0%) (chi-square statistic 8.85, Cramér's V = 0.55, 95% CI [0.17, 0.71]). PWH with CP used analgesics and adjunctive pain medications for CP more often than PWoH with CP (“Almost never” 12.5%, “Once in a while” 29.2%, “About half the time” 4.17%, “Frequently,” 12.5%, “Almost Always” 41.7% versus 80%, 20%, 0%, 0%; Cramér's V = 0.61 [95% CI 0.146, 0.80]). Participants with CP more frequently used opioids (48.3%) than those without CP (20.6%) (OR = 3.6, 95% CI [1.19, 10.8]).

### Chronic pain sources

**Fig. 1:**
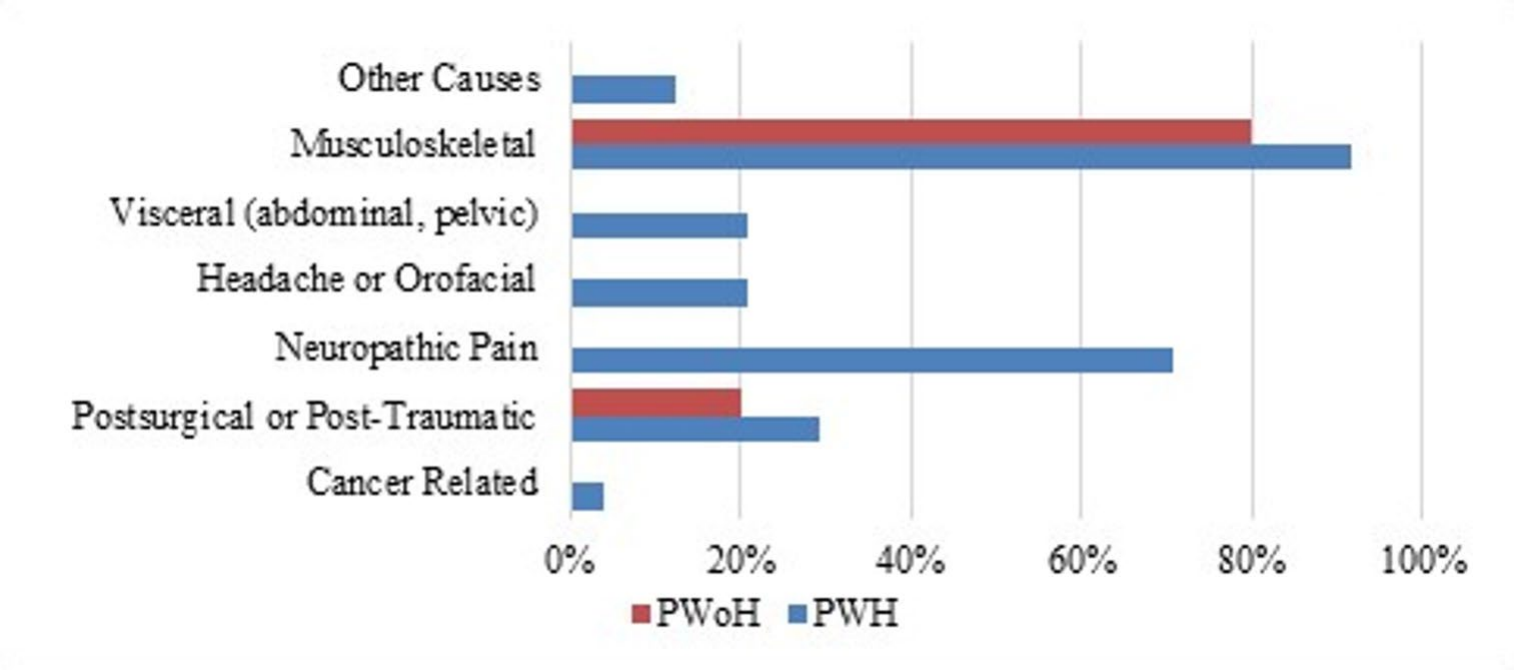
Self-reported sources of CP in people living with and without HIV Note. PWH = people with HIV; PWoH = people without HIV.

**Fig. 2:**
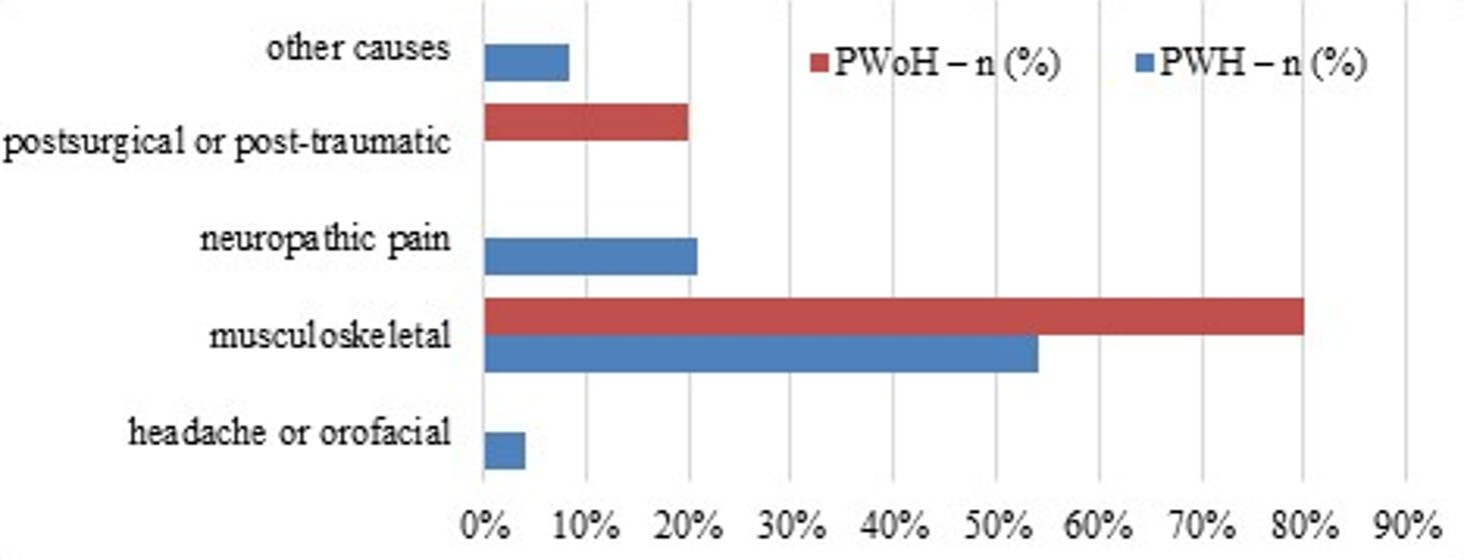
Self-reported primary source of pain in people living with versus without HIV. Note. PWH = people with HIV; PWoH = people without HIV.

Figure 1 shows the sources of CP reported by study participants for PWH and PWoH. Note that participants could report multiple pain sources; among PWH with CP, 79.2% reported multiple sources. Neuropathic pain was the only source category that differed between the two groups, affecting 70.8% of PWH versus none of the 5 PWoH meeting CP criteria; however, clinician-assessed distal neuropathic pain was present in 4.35% of PWoH overall (Supplemental Table 2). In both groups, musculoskeletal pain was the most frequent (80.0% versus 91.7%). The distribution of the other sources did not differ significantly between the two groups. Among PWH, the frequency of headache or orofacial pain was low, with only one individual affected, accounting for 4.2% of the cohort. Figure 2 illustrates the participant-reported *primary* source of pain among those with and without HIV. Neuropathic pain was the primary source in 5 PWH (20.8%), while other causes of pain were noted in only 2 participants (8.3%). Detailed summary of neuropathic signs and symptoms are provided in the Supplemental Material and summarized in Supplemental Table 2. Postsurgical or post-traumatic pain was reported as the primary source in none of the PWH CP group participants (vs 20% of their PWoH counterparts). PWoH reported no cases of a primary source of CP being headache or orofacial, neuropathic, or other causes. Musculoskeletal pain was reported as the primary source in 13 PWH (54.2%) versus 80% of the PWoH group.

### Relationship of chronic with depressed mood

**Fig. 3:**
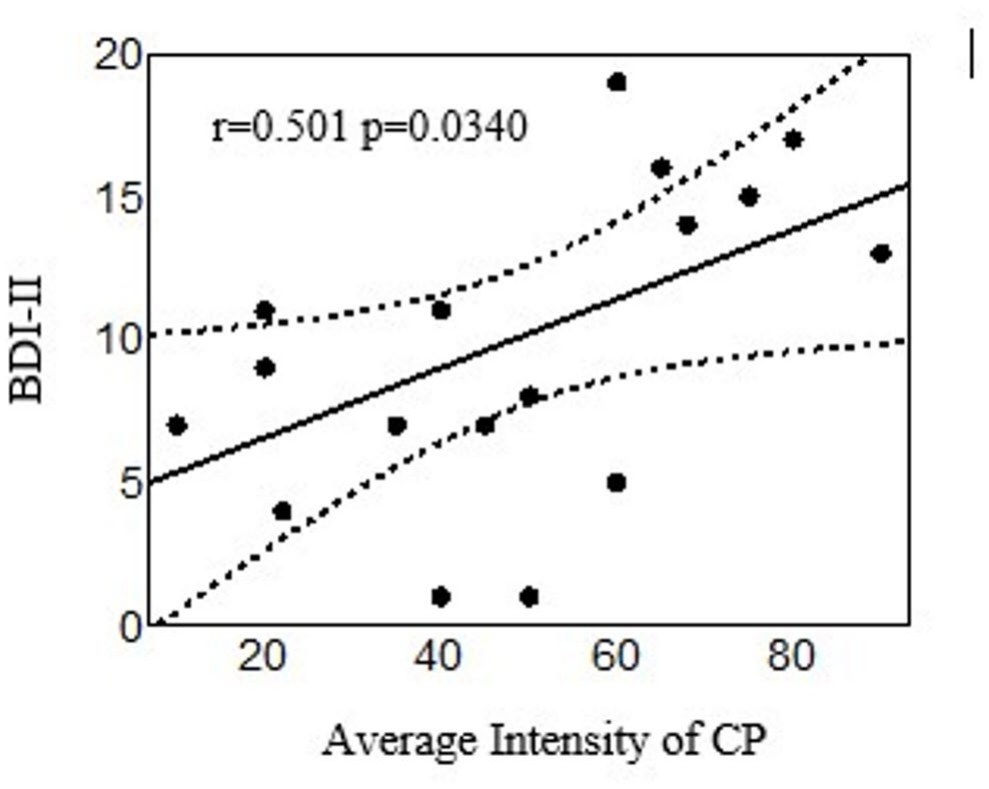
Relationship of CP to depressed mood (total BDI-II score) in people living with HIV. Note. BDI-II = Beck Depression Inventory version 2, CP = chronic pain.

Figure 3 shows the relationship between CP and depressed mood, indexed by the total BDI-II score, in PWH. PWoH are omitted due to the small sample size. BDI-II scores were missing for 25 participants because the data were collected remotely during the COVID-19 epidemic when ethical considerations dictated that participants were not asked about suicidal ideation if immediate in-person clinician intervention was not available. Given the small number of PWoH with both CP and BDI-II scores (n = 3), analyses of depression focused on PWH only. In PWH for whom data were available, BDI-II scores were significantly higher (worse depressed mood) in those with CP compared to those without (7.77 ± 5.86 vs 2.92 ± 3.06, d = 1.24). Increasing pain severity correlated with worse depressed mood in PWH (r = 0.501). Antidepressants were used by 45.0% of PWH; the association of CP with antidepressant use was weak (OR = 1.28, 95% CI [0.45, 3.61]). Table 3 shows effect sizes for the relationships of CP with the BDI-II subscales for PWH. PWoH were omitted due to the small sample size. Effect sizes in PWH for the somatic, affective, apathy, and anhedonia subscales were moderate in size (Cramér’s Vs range from 0.36 to 0.41). Bootstrapped 95% CIs (10,000 iterations) excluded zero for the BDI-II total score and for the somatic, affective, apathy, and anhedonia subscales; only the cognitive subscale CI included zero (Supplemental Table 3).

**Table 3:** Comparison of Beck Depression Inventory II subscales by CP status for people living with HIV.

HIV serostatus	BDI-II subscale	Cramér’s V CP versus no CP	FDR *p*
**PWH**	**Somatic**	**0.391**	**0.003**
**PWH**	**Affective**	**0.406**	**0.014**
**PWH**	**Apathy**	**0.368**	**0.014**
**PWH**	**Anhedonia**	**0.357**	**0.014**
PWH	Cognitive	0.288	0.198
PWoH	Somatic	0.094	0.993
PWoH	Affective	< 0.001	0.999
PWoH	Apathy	0.065	0.993
PWoH	Anhedonia	0.074	0.993
PWoH	Cognitive	0.260	0.993

*BDI-II* Beck Depression Inventory version 2, *FDR* false discovery rate multiple test correction, *PWH* people with HIV. PWoH rows are included for completeness but should be interpreted with caution given the very small number with available BDI-II data. Bolded rows represent statistically significant differences. Rows are ranked by significance values.

### Relationships between CP and other clinical measures

Among PWH, CP was associated with worse physical (37.5 ± 9.88 versus 52.5 ± 9.73, d = 1.17) but not mental health-related QOL (MOS) scores (48.1 ± 8.17 versus 55.0 ± 6.45, d = 0.55). PWH had more IADL complaints than PWoH (median [IQR] 1 [0, 2] versus 0 [0, 0], d = 0.729), but among PWH, those with and without CP did not differ in IADL complaints (1 [0.75, 4.25] versus 1 [0, 2], d = 0.362). PWH had higher (worse) PSQI scores than PWoH (8.78 ± 3.63 versus 6.84 ± 2.71, d = 0.563). Across all participants, PSQI scores were worse among those with CP than those without (9.52 ± 3.48 versus 6.83 ± 2.92, d = 0.78). In a multivariable model including both HIV serostatus and CP, only CP was significantly associated with PSQI (partial ηp^2^s for HIV serostatus, CP, and their interaction, respectively, 0.03, 0.08, and 0.01). PWH with CP had numerically but non-significantly lower (worse) mean NIH Toolbox Social Satisfaction Summary T Scores (41.3 ± 13.4 versus 47.9 ± 13.8; d = 0.49). In a multivariable analysis of the effects of CP and depressed mood (BDI-II) on quality of life (QoL [MOS-HIV] in PWH) the interaction term was not significant (t Ratio 0.49). CP and depressed mood had an additive adverse effect on QoL (partial ηp^2^s = 0.12, 0.18; model R^2^ = 0.51).

Because the neurotoxic nucleoside reverse transcriptase inhibitors (NRTIs) stavudine (d4T) and didanosine (ddI) are established causes of painful distal sensory polyneuropathy (DSP), we examined whether prior exposure to these agents contributed to CP (particularly DNP) in our cohort. None of the 40 virally-suppressed PWH were taking neurotoxic NRTIs at the time of evaluation; however, 16 (40.0%) reported past exposure. Prior exposure was associated with a higher likelihood of at least one objective examination finding consistent with DSP (78.6% versus 51.3%; OR = 3.48, 95% CI [0.84, 14.5]) and a nonsignificant elevation in DNP frequency (50.0% versus 31.9%; OR = 2.13, 95% CI [0.667, 6.78]). CP itself was also somewhat more common among previously exposed individuals (56.3% versus 42.6%; OR = 1.74, 95% CI [0.55, 5.45]). Among those with CP, DNP was reported by 66.7% of neurotoxic NRTI–exposed participants compared with 55.0% of those without such exposure (OR = 1.64, 95% CI [0.317, 8.45]).

PWH had a higher odds of polypharmacy than PWoH (OR = 41.8, 95% CI [7.13, 808]). In PWH, CP was not related to polypharmacy (OR = 1.46, 95% CI [0.381, 5.57]). While people with HIV showed higher rates of antidepressant medication use than PWoH (n = 18 [45%] versus n = 4 [17.4%], OR = 3.89, 95% CI [1.12, 13.5]); PWH with CP did not differ from those without CP (n = 10 [41.7%] versus n = 8 [50%], OR = 1.4, 95% CI [0.392, 5.00]).

### Impact of potential biases related to missing BDI-II data

Cramer’s V statistics for categorical variables comparing participants with vs. without BDI-II data were small (range 0.06–0.23; highest for CP status, V = 0.23). Effect sizes for continuous variables were modest (ds range from 0.05–0.60); nadir CD4 showed the largest difference (d = 0.60), with higher nadir CD4 in those missing BDI-II. CP rates trended higher in participants without BDI-II data (OR = 2.63 [0.91, 7.55]). Multivariable models adjusting for nadir CD4 further showed elevated CP rates in PWH for both those with (OR = 6.75, 95% CI [1.43, 31.8]) and without (OR = 3.38, 95% CI [0.52, 21.70]) BDI-II data. These results support robustness of the CP-depression associations to the missingness pattern.

## Discussion

This exploratory analysis in a small cohort suggests substantially higher CP frequency in virally-suppressed PWH compared to PWoH, generating hypotheses for future research. This elevated prevalence aligns with prior studies, reinforcing the unique vulnerability of PWH to CP due to biological, psychosocial, and clinical factors. Our findings confirm CP as a major burden in PWH, with clear detrimental effects on daily functioning, mood, and quality of life.

One key finding is the significantly higher frequency of neuropathic pain among PWH, which likely contributes to their overall CP burden. Neuropathic pain, primarily associated with DNP, was nearly absent in PWoH but prevalent in PWH. Neuropathic pain is known to be related to other adverse outcomes, such as depression and cognitive decline^33,40,41^. This difference underscores the role of HIV-related pathophysiology, including chronic immune activation and neuroinflammation, in the pathogenesis of neuropathic pain^42^. Neuropathic pain, in turn, was a key driver of activity interference in PWH. In our cohort, neuropathic pain was present in 70.8% of PWH with CP and in 0 of 5 PWoH meeting CP criteria, whereas PWoH predominantly reported musculoskeletal pain. Because neuropathic pain is typically more disabling and less responsive to first-line analgesics than musculoskeletal pain, this difference in pain type likely explains the greater interference with daily activities observed in PWH. If people without HIV but with neuropathic pain had been included, they might show similar activity interference. Our data support this interpretation—within PWH, neuropathic pain was strongly associated with activity interference, while HIV status alone was not. These findings suggest that targeted identification and treatment of neuropathic pain represents a key therapeutic priority for improving function in aging PWH. We found that historical exposure to neurotoxic NRTIs was associated with higher rates of DSP and DNP, suggesting increased vulnerability in these PWH. DNP accounted for a substantial proportion of CP symptoms in previously neurotoxic-NRTI-exposed PWH, although our sample size limits definitive inference.

The strong association between CP and depressed mood in PWH aligns with evidence for shared neurobiological pathways involving inflammation, neurotransmitter dysregulation, and altered neural circuitry^6,43–45^. Astrocytes and microglia contribute to mood and pain via responses to neuronal injury and neurotransmitter imbalance^46^, partly through NLRP3-mediated IL-1β and IL-18 signaling^47,48^. Both cell types show heightened activation in HIV^46^, and CP in PWH is linked to elevated pro-inflammatory M1 macrophage chemokines^49^. Together, these converging inflammatory and neurobiological processes likely intensify the bidirectional relationship between CP and mood disorders in virally-suppressed PWH, reinforcing the need for targeted research and intervention efforts on DNP in this population.

The CP and mood association observed in our cohort is further contextualized by a growing body of evidence linking psychosocial stigma to pain and mood outcomes in PWH. Intersectional HIV and chronic pain stigma has been independently associated with greater depressive symptom severity in PWH, with compounded stigma predicting greater mood burden than either stigma alone^50^. More recently, mixed evidence has emerged on whether enacted versus internalized HIV stigma is associated with pain severity, with sexual minority status independently predicting pain across samples^51^. A nationwide study of virally suppressed PWH in Denmark found that anticipated stigma was highly prevalent even in a well-resourced setting and was significantly associated with depression, anxiety, and social isolation, independent of clinical HIV factors^52^. Our study did not assess HIV or chronic pain stigma, and unmeasured stigma-related distress may have contributed to the CP-mood associations we observed. Future studies should incorporate validated multidimensional stigma assessments to disentangle social from biological determinants of CP and mood comorbidity in this population.

In addition to HIV-related immune activation, *inflammaging*—the chronic, low-grade inflammatory state associated with aging—may further increase vulnerability to CP in older PWH. Even with sustained viral suppression, many PWH demonstrate persistent elevations in inflammatory and immune-senescence markers, including monocyte/macrophage activation and glial activation in the CNS, which overlap with biological pathways driving peripheral and central sensitization and nociceptive signaling^44,47,49^. Inflammaging has been associated with accelerated multimorbidity and functional decline in aging populations, and PWH show evidence of an amplified form of this process compared to peers without HIV^44^. Given that our cohort reflects the increasingly older demographic of PWH, inflammaging may compound CP burden despite viral suppression, helping explain why pain remained highly prevalent and disabling in our sample. These observations support future studies integrating inflammatory biomarkers to clarify the interaction among aging, chronic inflammation, and pain mechanisms in PWH.

Overlapping neural pathways, such as the mesolimbic dopaminergic reward system, may help explain the link between CP and depressed mood. Dopamine deficiency, common in PWH, is associated with greater CP and anhedonia^53–55^. CP also disrupts the dlBNST-to-VTA pathway, where stress-related CRF signaling suppresses mesolimbic dopamine activity, contributing to depressed mood^55^. Serotonin (5-HT) and norepinephrine (NE) systems, which underlie both CP and mood regulation, mediate the effects of most antidepressants^56^. Because these neurotransmitters participate in endogenous analgesia, 5-HT–based antidepressants may reduce certain pain conditions^57,58^.

Our study also highlights significant impairments in physical health-related quality of life among PWH with CP, confirming previous findings^59,60^. CP in this cohort was associated with worse sleep quality and greater interference with daily activities. These findings emphasize the multidimensional impact of CP on physical, psychological, and social domains, necessitating integrated care approaches. The reliance on opioids and adjunctive pain medications among PWH with CP further underscores the challenges of pain management in this population. Given the risks of opioid dependence, adverse effects, and drug-drug interactions, multimodal pain management strategies tailored to the specific needs of PWH are imperative^5^.

Contrasting with PWoH, we found in PWH a high frequency of multiple concurrent comorbid sources of pain, including musculoskeletal, visceral (abdominal, pelvic), neuropathic pain, and postsurgical or post-traumatic^1–3,13^. This issue is important because treating CP in PWH requires considering these multiple sources and the need for multidisciplinary, integrated care.

Interestingly, demographic factors such as age and sex did not show significant associations with CP in PWH, diverging from patterns observed in the general population. This discrepancy may reflect the complex interplay of HIV-specific factors, including accelerated aging and unique comorbidity profiles. The lack of association between CP and HIV-specific clinical variables such as CD4 counts and ART status suggests that CP in PWH is influenced more by chronic immune activation and comorbid conditions than by direct virologic control.

### Limitations

As a pilot study, the relatively small sample sizes, particularly for PWoH (n = 23), limit statistical precision. The primary between-group odds ratio (OR = 5.4, 95% CI [1.67, 17.5]) illustrates this directly: while the point estimate is large, the wide confidence interval reflects genuine uncertainty about the true effect size, and readers should emphasize the direction and possible range of the effect rather than the precise estimate. This caveat applies throughout: wide confidence intervals on ORs and effect sizes reflect sample size constraints, not necessarily unreliable findings. Notably, our CP prevalence in PWoH (22%) aligns with established population estimates of 20–30%, and our PWH findings are consistent with prior literature^1–3,61^, supporting the representativeness of our samples despite their modest size.

The lack of association between CP and HIV-specific clinical variables should be interpreted cautiously. Our PWH cohort was uniformly virally suppressed with a relatively narrow nadir CD4 range, reducing variability and statistical power to detect associations even if they exist. Throughout the manuscript we therefore report effect sizes and confidence intervals alongside p-values, allowing readers to judge the magnitude and potential clinical importance of observed relationships independently of significance thresholds. Larger studies with broader representation of HIV disease severity will be needed to determine whether null findings reflect true absence of association or insufficient power.

Generalizability is limited by our single-center recruitment of a predominantly male (76%), virally suppressed, community-dwelling sample. The gender imbalance between PWH (24% female) and PWoH (45% female) reflects US HIV epidemic demographics^62^ but limits our ability to examine sex-specific effects. Because all PWH were virally suppressed, we could not evaluate the relationship between CP and ART adherence; findings should be interpreted in the context of a well-treated cohort, which may not generalize to global populations with greater immunologic compromise or incomplete virologic control. The exploratory nature of the analyses and absence of longitudinal data also constrain causal inference.

Residual confounding by unmeasured variables cannot be excluded. The primary focus of this pilot study was characterizing CP prevalence and its major clinical correlates in PWH; a comprehensive evaluation of medical comorbidities was beyond the scope of this initial work. Conditions such as hypertension, which may differ between PWH and PWoH and could contribute to pain differences, were not systematically examined here but represent an important target for future larger studies. Socioeconomic data were limited to education and employment; granular income and occupational data were not collected. Pain treatment history prior to enrollment was not systematically assessed. Future studies should comprehensively evaluate these potential confounders.

Regarding measurement, reliance on self-reported measures for pain and mood introduces potential biases, though use of validated instruments mitigates these. Participants’ self-reported pain sources may not always align with clinical physio-anatomical diagnoses, due to limited health literacy or referred pain; however, this does not detract from the core objective of capturing the patient’s lived experience of pain. BDI-II data were missing for 25 participants due to COVID-related ethical constraints on remote administration of suicidality items; sensitivity analyses indicated this was unlikely to meaningfully bias mood-related findings.

### Implications and future directions

Our findings emphasize the need for a comprehensive, multidisciplinary approach to CP management in PWH. Incorporating structured pain assessments into routine HIV care may help address the broad impact of CP on mood, pain perception, and social functioning. Evidence supports integrated strategies that combine pharmacologic and non-pharmacologic approaches, including physical activity, which may offer protective effects against CP. Regarding pharmacologic approaches, the observed reliance on analgesics in our study population indicates the importance of careful medication management to avoid adverse effects and interactions, particularly with antiretroviral therapies.

Future research should include larger, longitudinal studies to clarify causal pathways linking HIV, CP, and mood disorders, with inflammatory biomarkers and neuroimaging to identify underlying mechanisms and therapeutic targets. Evaluating multimodal interventions such as physical activity therapy and cognitive-behavioral therapy, and examining how social determinants contribute to CP and mood disorders, may inform more equitable care. Precision medicine strategies—such as genetic and epigenetic profiling—along with digital health tools for real-time monitoring, could support individualized treatment. Continued exploration of innovative options, including neuromodulation, may further enhance CP management in PWH.

## Supplementary Information

Below is the link to the electronic supplementary material.

Supplementary Material 1

Supplementary Material 2

## Data Availability

The data that were analyzed are available from the National NeuroAIDS Tissue Consortium-CHARTER Data Coordinating Center (https://www.nntc.org/content/relationship-charter) upon request. The code supporting the findings of this study is available upon reasonable request to the authors. Data and code can be requested by contacting Dr. Ronald J. Ellis at [roellis@health.ucsd.edu] (mailto:roellis@health.ucsd.edu).
