# Supplementary material for: Chronic pain in a modern virally suppressed HIV cohort is associated with disability and poorer mental health
Source: Sci Rep. 2026 May 13;16:21886. doi: 10.1038/s41598-026-52912-x (PMC13365815; doi:10.1038/s41598-026-52912-x)
Supplement: Supplementary file 1 — Supplementary Material 1 [file 41598_2026_52912_MOESM1_ESM.docx]

**REVISED SUPPLEMENTAL MATERIAL**

*Chronic Pain in a Modern Virally Suppressed HIV Cohort: Associations with Medical Characteristics, Mental Health, and Disability*

**Supplemental Methods**

**Chronic pain questionnaire**

To evaluate chronic pain and its relation to depressed mood and functional outcomes, we administered a questionnaire that operationalizes the IASP/ICD-11 diagnostic criteria. While not formally validated, it directly maps to IASP classifications for: (1) pain duration and frequency, (2) pain intensity and interference, (3) pain source categories, and (4) severity specifiers. The complete questionnaire is provided in Supplementary Appendix A to facilitate replication and future validation studies. Chronic pain was defined as daily or almost daily pain lasting over 3 months. Those who reported chronic pain answered additional questions about the intensity of their pain, interference in daily activities, use of pain medications, and pain source.

**Depressed mood and other psychiatric characteristics**

Current mood symptoms were evaluated with the Beck Depression Inventory-II (BDI-II), a 21-item self-report instrument rated on a 4-point Likert scale. BDI-II total score ranges from 0 to 63. Component BDI-II subscales capture cognitive, somatic, affective, and apathy symptoms. Anxiety was characterized by the Overall Anxiety Severity and Impairment Scale (OASIS). Lifetime psychiatric and substance disorders were characterized using the Composite International Diagnostic Interview (CIDI).

**Medical characteristics**

HIV disease was diagnosed by enzyme-linked immunosorbent assay with Western blot confirmation. Routine clinical chemistry panels, complete blood counts, and CD4+ T cells (flow cytometry) were performed. Levels of HIV viral load in plasma were measured using reverse transcriptase-polymerase chain reaction (Amplicor, Roche Diagnostics), with a lower limit of quantitation of 50 copies/mL. HIV viral load was dichotomized as detectable vs. undetectable at the LLQ of 50 copies/mL. CD4 nadir was assessed by self-report. All participants completed a substance use history. All participants underwent urine toxicology testing for substances including opioids, amphetamines, cannabis, and cocaine. Opioid use was evaluated through self-report and urine toxicology; use was classified as present if either was positive. Detailed medical and neurological histories, concomitant medications, and antiretroviral (ARV) drug exposure history were captured via a structured, clinician-administered questionnaire.

**Clinical assessment of neuropathy and neuropathic pain**

Because sensory polyneuropathy and neuropathic pain are common sources of chronic pain in PWH, we performed targeted interviews and neurological examinations to characterize these. Centrally trained clinicians conducted standardized, validated evaluations including clinical examination for neuropathy signs (bilateral distal vibration, sharpness, and touch loss in the legs and feet, and reduced ankle reflexes). Self-reported distal neuropathic pain (DNP) was defined as burning, aching, or shooting symptoms in the distal legs and feet and was classified into five grades of clinician-rated pain severity based on participant reports: none, slight (occasional, fleeting), mild (frequent), moderate (frequent, disabling), and severe (constant, daily, disabling, requiring analgesic medication or other pain medication).

**Quality of life**

Quality of life was assessed using the Medical Outcomes Study HIV Health Survey Short Form 36 (MOS-HIV SF-36), a reliable and valid tool for assessing overall quality of life, daily functioning, and physical health. The MOS-HIV contains 36 questions that assess various physical and mental dimensions of health. Items are grouped into two categories (Physical and Mental Health), with 9 subcategories, and Overall QoL.

**Sleep**

The Pittsburgh Sleep Quality Index (PSQI) is a self-rated questionnaire that assesses sleep quality and disturbances over 1 month. It consists of 19 items combined to form seven component scores. Each component is scored on a scale from 0 to 3, with 3 indicating the greatest dysfunction. The seven component scores are summed to produce a global PSQI score (range 0 to 21, higher scores indicating worse sleep quality).

**Social functioning**

The NIH Toolbox Social Satisfaction Factor Score measures aspects of social relationships, including emotional support, instrumental support, friendship, loneliness, and perceived rejection. This summary score represents an individual's overall social satisfaction, with higher scores indicating greater satisfaction.

**Activities of daily living**

Instrumental activities of daily living (IADLs) were evaluated using an adaptation of the Lawton-Brody IADL scale that assessed self-reported changes in levels of independence in performing 16 everyday tasks. Each task is scored as 0 (no change from best functioning) or 1 (more dependent now). The IADL total score ranges from 0 to 16.

**Supplemental Results**

**Neuropathy signs**

Contingency table analyses were performed to evaluate the relationship between HIV serostatus and signs of peripheral neuropathy. Prevalence rates for all three clinical signs were higher in PWH than in PWoH, with diminished or absent deep tendon reflexes showing a statistically significant association. Reflex impairment was present in 45.5% of PWH compared to 10.5% of PWoH, with a Pearson p-value of 9.70e-3. The odds ratio for reflex impairment in PWH was 7.08 (95% CI: 1.41, 35.7), indicating that PWH had over seven times the odds of exhibiting diminished reflexes compared to PWoH. While not statistically significant, other modalities also showed higher prevalence in the PWH group. Impaired pinprick sensation was noted in 27.3% of PWH versus 10.5% of PWoH (p = 1.55e-1), with an odds ratio of 3.19 (95% CI: 0.610, 16.7). Impaired vibration perception was found in 50.0% of PWH and 36.8% of PWoH (p = 3.56e-1), corresponding to an odds ratio of 1.71 (95% CI: 0.543, 5.41).

These findings demonstrate that while all three clinical signs of neuropathy were more prevalent in PWH, the loss of deep tendon reflexes serves as the most robust differentiator by serostatus in this cohort. The significant elevation in reflex impairment, with an odds ratio of 7.08, suggests that large-fiber dysfunction or root involvement remains a prominent feature in the clinical presentation of HIV-associated neurological damage. The lack of statistical significance for vibration and pinprick sensation, despite higher raw frequencies in PWH, may reflect limited power to detect more subtle differences in small-fiber or dorsal column function within this specific sample size.

**Neuropathy symptoms**

Subjective symptoms also differed significantly by serostatus. Distal neuropathic pain was reported by 55.0% of PWH compared to only 4.35% of PWoH (p < 1.00e-4). Similarly, paresthesias were present in 57.5% of PWH versus 13.6% of PWoH (p = 0.0065) and reported loss of sensation affected 42.5% of PWH compared to 8.70% of PWoH (p = 0.0333).

**Supplemental Table 1.** HIV-related clinical correlates of chronic pain in persons living with HIV (PWH).

| **Predictor** | **OR** | **95% CI** | **p-value** | **Model** |
| --- | --- | --- | --- | --- |
| **Univariable Models** | | | | |
| Current CD4 (per 100 cells/uL) | 0.95 | [0.74, 1.22] | 0.68 | Univariable |
| Nadir CD4 (per 100 cells/uL) | 0.89 | [0.62, 1.28] | 0.53 | Univariable |
| HIV duration (per 5 years) | 1.08 | [0.76, 1.53] | 0.67 | Univariable |
| **Multivariable Model** | | | | |
| Current CD4 (per 100 cells/uL) | 0.97 | [0.73, 1.28] | 0.82 | Multivariable |
| Nadir CD4 (per 100 cells/uL) | 0.91 | [0.60, 1.38] | 0.66 | Multivariable |
| HIV duration (per 5 years) | 1.10 | [0.74, 1.64] | 0.63 | Multivariable |
| Note. OR = odds ratio; CI = confidence interval. Multivariable model adjusted for age and sex. | | | | |

**Supplemental Table 2.** Prevalence of neuropathy signs and symptoms in people with HIV (PWH) and people without HIV (PWoH).

| **Parameter** | **PWH Prevalence (n)** | **PWoH Prevalence (n)** | **OR** | **Lower 95% CI** | **Upper 95% CI** | **p** |
| --- | --- | --- | --- | --- | --- | --- |
| **Reflexes (Diminished)** | 45.5% (15/33) | 10.5% (2/19) | 7.08 | 1.41 | 35.70 | 0.0097 |
| **Pin (Sharp Sensation)** | 27.3% (9/33) | 10.5% (2/19) | 3.19 | 0.61 | 16.70 | 0.155 |
| **Vibration (Tuning Fork)** | 50.0% (17/34) | 36.8% (7/19) | 1.71 | 0.54 | 5.41 | 0.356 |
| **Distal Neuropathic Pain** | 55.0% (22/40) | 4.35% (1/23) | 26.89 | 3.30 | 219.30 | <.0001 |
| **Paresthesias** | 57.5% (23/40) | 13.6% (3/22) | 8.57 | 2.18 | 33.70 | 0.0065 |
| **Loss of Sensation** | 42.5% (17/40) | 8.70% (2/23) | 7.76 | 1.60 | 37.70 | 0.0333 |
| Note. CI = confidence interval; OR = odds ratio; PWH = people with HIV; PWoH = people without HIV. Wide confidence intervals are due to small sample size and should be considered with caution. | | | | | | |

**Supplemental Table 3. Bootstrapped 95% confidence intervals for BDI-II effect sizes by chronic pain status (CP vs. no CP) in people living with HIV (PWH).**

| **Subscale** | ***d*** | **95% CI Lower** | **95% CI Upper** | **Significant** |
| --- | --- | --- | --- | --- |
| BDI-II Total | -1.49 | -2.53 | -0.83 | Yes |
| Somatic | -1.66 | -3.06 | -0.88 | Yes |
| Affective | -1.21 | -2.19 | -0.54 | Yes |
| Apathy | -1.32 | -2.53 | -0.50 | Yes |
| Anhedonia | -1.33 | -2.75 | -0.53 | Yes |
| Cognitive | -0.56 | -1.30 | 0.22 | No |

Note. Effect size estimates (mean differences, CP vs. no CP) are presented with bootstrapped 95% confidence intervals (10,000 iterations). Negative values indicate higher (worse) BDI-II scores in the chronic pain group. Confidence intervals that exclude zero indicate robust associations. BDI-II = Beck Depression Inventory version 2; CI = confidence interval; CP = chronic pain; PWH = people with HIV. FDR-corrected p-values are reported in Table 3 of the main manuscript.
